# The Effects of Pregnancy on Amino Acid Levels and Nitrogen Disposition

**DOI:** 10.3390/metabo13020242

**Published:** 2023-02-07

**Authors:** Luke F. Enthoven, Yuanyuan Shi, Emily E. Fay, Sue Moreni, Jennie Mao, Emma M. Honeyman, Chase K. Smith, Dale Whittington, Susan E. Brockerhoff, Nina Isoherranen, Rheem A. Totah, Mary F. Hebert

**Affiliations:** 1 Department of Pharmacy, University of Washington, Seattle, WA 98195, USA; 2 Department of Medicinal Chemistry, University of Washington, Seattle, WA 98195, USA; 3 Department of Obstetrics and Gynecology, University of Washington, Seattle, WA 98195, USA; 4 Department of Biochemistry, University of Washington, Seattle, WA 98195, USA; 5 Department of Pharmaceutics, University of Washington, Seattle, WA 98195, USA

**Keywords:** metabolomics, ammonia-urea cycle, arginine, pregnant, postpartum, pathway, amino acid, branched chain amino acid

## Abstract

Limited data are available on the effects of pregnancy on the maternal metabolome. Therefore, the objective of this study was to use metabolomics analysis to determine pathways impacted by pregnancy followed by targeted confirmatory analysis to provide more powerful conclusions about metabolic alterations during pregnancy. Forty-seven pregnant women, 18–50 years of age were included in this study, with each subject serving as their own control. Plasma samples were collected between 25 and 28 weeks gestation and again ≥3 months postpartum for metabolomics analysis utilizing an HILIC/UHPLC/MS/MS assay with confirmatory targeted specific concentration analysis for 10 of the significantly altered amino acids utilizing an LC/MS assay. Principle component analysis (PCA) on metabolomics data clearly separated pregnant and postpartum groups and identified outliers in a preliminary assessment. Of the 980 metabolites recorded, 706 were determined to be significantly different between pregnancy and postpartum. Pathway analysis revealed three significantly impacted pathways, arginine biosynthesis (*p* = 2 × 10^−5^ and FDR = 1 × 10^−3^), valine, leucine, and isoleucine metabolism (*p* = 2 × 10^−5^ and FDR = 2 × 10^−3^), and xanthine metabolism (*p* = 4 × 10^−5^ and FDR = 4 × 10^−3^). Of these we focused analysis on arginine biosynthesis and branched-chain amino acid (BCAA) metabolism due to their clinical importance and interconnected roles in amino acid metabolism. In the confirmational analysis, 7 of 10 metabolites were confirmed as significant and all 10 confirmed the direction of change of concentrations observed in the metabolomics analysis. The data support an alteration in urea nitrogen disposition and amino acid metabolism during pregnancy. These changes could also impact endogenous nitric oxide production and contribute to diseases of pregnancy. This study provides evidence for changes in both the ammonia-urea nitrogen and the BCAA metabolism taking place during pregnancy.

## 1. Introduction

Pregnancy induces broad metabolic and physiological changes. Among these are changes in endocrine signaling, nitrogen disposition, amino acid metabolism, protein metabolism, vascular resistance, and glucose metabolism [1]. While most of the biological changes that take place during pregnancy are known, the biochemical mechanisms by which these changes are achieved and how they can contribute to diseases of pregnancy are relatively unknown. Many of the metabolic alterations that occur during pregnancy are to support the protein needs of the fetus through changes in amino acid metabolism and the linked nitrogen disposition [2,3,4,5]. Several studies examined the changes in plasma concentrations of amino acids and other metabolites during pregnancy as well as amino acid catabolism, but none have applied a broad metabolomics approach with quantitative confirmational analysis [3,4,5,6,7,8].

In humans, the ammonia-urea cycle is the primary method for toxic nitrogen removal using amino acid (AA) intermediates, most notably ornithine, citrulline, and arginine [2]. Urea synthesis, clearance, and the concentrations of some ammonia-urea cycle AA intermediates have been altered by pregnancy [3,4,5]. The essential AAs valine, leucine, and isoleucine are hydrophobic, branched-chain amino acids (BCAAs) that make up about 20% of the proteins consumed in a standard Western diet. BCAAs are vital for protein structure and various aspects of metabolism [9]. BCAAs are deaminated by branched chain amino transferase (BCAT) enzymes, forming 𝛼-keto acids that are then dehydrogenated and enter the Krebs cycle as energy producing intermediates [9,10]. These AAs have been implicated in fetal growth through their relationship with the expression of insulin growth factors [11]. The objective of this study was to determine the specific impact of normal pregnancy on the maternal metabolome. This was achieved by utilizing a broad metabolomics and pathways analysis followed by targeted confirmational quantitative analysis to increase the power of metabolomics and strengthen findings.

## 2. Materials and Methods

### 2.1. Study Participants

All enrolled participants were between 18 and 50 years of age and were CYP2D6 extensive metabolizers based on the genotype originally selected to take part in a larger study assessing the effect of retinoids on CYP2D6 enzyme activity in pregnancy [12]. Subjects provided written informed consent to participate in this study. The study protocol was approved by the institutional review board at the University of Washington and conducted in accordance with its guidelines.

Each woman was considered generally healthy at the time of enrollment. Participants were excluded for fever, cough, known kidney or liver disease, diabetes, body mass index (BMI) > 30 kg/m^2^, and psychiatric illnesses requiring medication. A significant number of medications served to disqualify participants from the study. A list of medications can be found in Supplementary File S3. On the day of the study, participants were evaluated with a comprehensive metabolic panel performed by University of Washington Medical Center Laboratory Medicine. Pregnant women were recruited from the University of Washington Medical Centers and Clinics.

Participants were asked to record all dietary intake (food and beverages) on the three days leading up to each study day. Average daily protein consumption for each subject’s dietary intake was determined utilizing Fooducate Ltd. (San Francisco, CA, USA).

Each subject completed three study days but only samples from study day 1 (sd1) and study day 3 (sd3) were used in this analysis. Sd1 took place during pregnancy (25–28 weeks gestation) and sd3 took place postpartum (≥3 months). A single blood sample from each subject was collected on each study day, at approximately 10:00 a.m. and approximately 1 h following the start of breakfast. This sampling window was chosen in accordance with the needs of the larger study on CYP2D6 metabolism [12]. Blood was collected into EDTA vacutainer tubes, placed on ice and then the plasma separated via centrifugation at 4 °C. The plasma samples were then immediately frozen at −80 °C until metabolomic analysis. Urine was collected over a 4-h period, and creatinine clearance was estimated using CrCL = [(Uv)(UCr]/[(SCr)(Time)], where Uv is urine volume, UCr is urine creatinine, and SCr is serum creatinine. Subjects’ height, weight, age, and weeks pregnant or postpartum were recorded along with current medical conditions and obstetric history. Each subject served as her own control (Table 1).

### 2.2. Metabolomics Analysis

Metabolomics analysis was carried out by Metabolon Inc. adapted from previously described methods [13]. Plasma samples were prepared using the automated MicroLab STAR system from Hamilton Company, and subject to recovery standards for quality control. Protein removal, small molecule dissociation from protein, and recovery of chemically diverse metabolites were achieved by precipitation with methanol under vigorous shaking for 2 min followed by centrifugation. Resulting extracts were divided into five fractions: two for analysis by two separate reverse phase ultra performance liquid chromatography with tandem mass spectrometry (UPLC/MS/MS) methods with positive ion electrospray ionization (ESI), one for analysis by reverse phase (RP)/UPLC/MS/MS with negative ESI, one for analysis by HILIC/UPLC/MS/MS with negative ion mode ESI, and one reserved for backup. Samples were placed briefly on a TurboVap^®^ (Zymark) to remove the organic solvent. The sample extracts were stored overnight under nitrogen before preparation for analysis.

A global biochemical profiling analysis was used to analyze the extracted samples and employed four unique arms. In the first arm, samples were analyzed using reverse phase chromatography and eluted using a C18 column (Waters UPLC BEH C18 − 2.1 × 100 mm, 1.7 µm) with a 0.35 mL/min flow rate and water (mobile phase A) and methanol (mobile phase B) mobile phases optimized for hydrophilic compounds (0.05% perfluoropentanoic acid (PFPA) and 0.1% formic acid, pH~2.5, LC/MS Pos Polar). LC/MS Pos Polar utilized a linear gradient from 5% mobile phase B to 80% mobile phase B over 3.35 min.

The second arm samples were also analyzed utilizing reverse phase chromatography and the same C18 column as the first arm with a 0.60 mL/min flow rate and water (mobile phase C) and 50% methanol with 50% acetonitrile (mobile phase D) mobile phases optimized for hydrophobic compounds (0.05% PFPA and 0.1% formic acid, pH~2.5, LC/MS Pos Lipid). LC/MS Pos Lipid utilized a linear gradient from 40% mobile phase D to 99.5% mobile phase D over 1.0 min, hold 99.5% phase D for 2.4 min. The metabolites in the first two arms were detected using positive ion electrospray ionization.

The third arm utilized the same C18 column with a 0.35 mL/min flow rate, this time including water (mobile phase E) and 95% methanol with 5% water (mobile phase F) mobile phases (6.5 mM ammonium bicarbonate, pH 8, LC/MS Neg). LC/MS Neg utilized a linear gradient from 0.5% mobile phase F to 70% mobile phase F over 4.0 min followed by rapid gradient to 99% phase F in 0.5 min. Analytes were detected using negative ion electrospray ionization conditions.

The fourth arm used a hydrophobic interaction chromatography column (Waters UPLC BEH Amide 2.1 × 150 mm, 1.7 µm) with a 0.5 mL min flow rate, this time with 15% water, 5% methanol, and 80% acetonitrile (mobile phase G) and a 50% water and 50% acetonitrile (mobile phase H) mobile phases (10 mM ammonium formate, pH 10.8, LC/MS Polar). LC/MS Polar utilized a linear gradient from 5% mobile phase H to 50% mobile phase H over 3.5 min followed by a linear gradient from 50% phase H to 95% phase H in 2 min. Analytes were detected using negative ion monitoring on electrospray mode using the mobile phase. For all methods the MS analysis alternated between full scan MS and data dependent MS*^n^* scans, generally covering 70–1000 *m/z.*

Metabolites of interest were identified by automated comparison of features in the experimental samples to a reference library that included retention time, molecular weight (*m/z*), preferred adducts, in-source fragments, and associated MS spectra for purified standards. Curation by visual inspection for quality assurance and peak identification was conducted using software developed at Metabolon Inc. Peaks were quantified using area-under-the-curve from MS/MS. Due to the analysis spanning multiple days, data normalization to a median of one (1.00) for each metabolite instrument batch was performed to correct any day-to-day instrumental variation.

Quality control and data curation were performed to ensure that identification of metabolites was consistent and true. Data identified as background noise and system artifacts were removed.

### 2.3. Confirmational Liquid Chromatography Mass Spectrometry

Confirmational analysis was performed using liquid chromatography mass spectrometry (LC/MS) through a targeted assay and quantitation for proline, valine, threonine, leucine, isoleucine, arginine, orthinine, citrulline, homoarginine, and homocitrulline. Standards for proteinogenic amino acids (proline, valine, threonine, leucine, and isoleucine) were prepared using a 2.5 mM mix of proteinogenic amino acids. The other amino acid standards (arginine, orthinine, citrulline, homoarginine, and homocitrulline) were prepared using dry powder stocks.

Proline, valine, threonine, leucine, isoleucine, and arginine samples were prepared using 50 μL internal standard mixed with 2 μL serum followed by protein precipitation with 450 μL acetonitrile. Samples were then centrifuged at 18,000× *g* for 10 min followed by LC/MS analysis.

A Waters BEH Amide 2.1 × 100 column was used with positive electrospray ionization to achieve separation. The A mobile phase consisted of 0.075% formic acid in 20 mM ammonium acetate, and 30% acetonitrile. The B mobile phase consisted of 0.075% formic acid in 20 mM ammonium acetate, and 93% acetonitrile. Both were run over 11 min with a flow rate of 0.3 mL/min. The limit of detection for each compound was as follows; proline 0.0031 μM, valine 0.0031 μM, threonine 0.0015 μM, leucine 0.004 μM, isoleucine 0.0066 μM, and arginine 0.165 μM. Limit of quantification was equal to the limit of detection for an analyte plus two standard deviations or the lowest calibration point.

Citrulline, ornithine, homoarginine, and homocitrulline samples were prepared using 60 μL internal standard mixed with 40 μL serum followed by protein precipitation with 300 μL acetonitrile. Samples were then centrifuged at 18,000× *g* for 10 min followed by LC/MS analysis.

A SeQuant ZIC-HILIC, 20 × 2.1 mm, 3.5 µm, 100 Ȧ column was used. The A mobile phase consisted of 20 mM ammonium acetate, 95% acetonitrile, and 5% H_2_O. The B mobile phase consisted of 20 mM ammonium acetate, 50% acetonitrile, and 50% H_2_O. Both were run over 10.5 min with a flow rate of 0.2 mL/min. The limit of detection for each compound was as follows; ornithine 5.1 × 10^−4^ pM, citrulline 3.9 × 10^−4^ pM, homoarginine 3.6 × 10^−4^ pM, and homocitrulline 3.6 × 10^−4^ pM. The limit of quantification was equal to the limit of detection for an analyte plus two standard deviations or the lowest calibration point.

Standard curves were generated from 6.93 μM to 1.00 nM of unlabeled amino acids and used to determine concentrations of unknown samples. These curves were weighted 1/x with accuracy ensured by separate quality control samples.

Resulting sample data quantitation was subjected to statistical analysis and compared to the metabolomics data to confirm the significance of the metabolomics findings.

### 2.4. Statistical Analysis

Pregnant and postpartum samples for both metabolomics and confirmational analysis were compared using a paired Student’s *t*-test where $t=\frac{{\bar{x}}_{1}-{\bar{x}}_{2}}{\left(\frac{Sd}{\sqrt{n}}\right)}\;$, with *n* − 1 degrees of freedom, where ${\bar{x}}_{1}$, ${\bar{x}}_{2}$ are the sample means for pregnant and postpartum study days, respectively, *S*_d_ is the standard deviation of the differences, *n* is the number of subjects (so there are 2*n* observations).

After *p*-values were determined and metabolites sorted by *p*-value from the paired Student’s *t*-test, a q-value was used to estimate the adjusted type one error rate for multiple comparisons. Q-values were generated for all pregnant and postpartum comparisons. BioConductor q-value function in R Studio was used to determine q-values from *t*-test *p*-values where $Q_{j}=Q\left(P_{j}\right)=minFDR{\left(t\right)}_{t\geq P_{j}}$ [14]. FDR in this case refers to the ratio of false positives to total positive results. For individual metabolites a *p*-value cutoff of *p* < 0.05 and q value cutoff of q < 0.01 were used to determine significant metabolites while accounting for both type 1 error and false discovery rate.

Confirmatory analysis encompassed far fewer compounds, decreasing the need for a measure of FDR. For this smaller data set, a Bonferroni correction was used to correct for multiple comparisons.

Principle component analysis (PCA) and orthogonal partial least square-discriminant (O-PLS-DA) analysis were used as unsupervised analysis to reduce the dimension of the data and display separation between the two groups. This was a preliminary analysis to determine outliers and variation between the study days, but the displayed separation may be due to components unrelated to the pregnancy and postpartum status. The quality of both models was assessed with the parameters R^2^Y and Q^2^ (>0.85). PCA and O-PLSDA analysis were performed using R Studio.

For preliminary analysis, significance for metabolite alterations between pregnancy and postpartum was defined as *p* < 0.1 and q < 0.1. After PCA and O-PLSDA preliminary analysis, significance for individual metabolites was defined as *p* < 0.05 and q < 0.01. Significance for pathways was defined as *p* < 0.05 and FDR < 0.1. A threshold minimum fold change of 2 was also applied for metabolites being considered in pathways analysis.

### 2.5. Pathways Analysis

Peak area values were normalized for each metabolite by dividing the raw peak area values by the median value for that metabolite in each instrument batch. This gives each batch and consequently metabolites, a median of one. The data were also imputed in order to preserve the statistical power of our analysis. Imputation included treating missing values as informative blanks and replacing them with the minimum observed value in the normalized peak area data.

MetaboAnalyst 5.0 (http://metaboanalyst.ca) was used to perform joint pathway analysis [15]. Differential metabolites identified in Metaboanalyst peak area data were imported as HMDB ID. Inclusion criteria for metabolites in pathways analysis was a false discovery rate (FDR) < 0.1 and a minimum two-fold change between groups. The metabolomic pathways considered were from the KEGG database. Metaboanlayst then performed a one-way analysis of variance and correlation analysis to identify significant compounds to contrast with significant compounds identified with the paired Student’s *t*-test.

Fisher’s exact test and degree centrality were utilized to determine pathway enrichment, resulting in pathway weighted and FDR adjusted *p*-values [15,16]. Impact scores were calculated. Impact scores represent the cumulative percentage of the degree of centrality based on degree centrality algorithms. In this case, each node considered is a metabolite and degree centrality represents the number of links between each node. More connected nodes are noted with higher degree centrality and therefore higher impact scores, helping to determine the importance of metabolic alterations in the KEGG arginine biosynthesis pathway.

Pathway *p*-values were subject to a Holm adjustment and an FDR was calculated by Metaboanalyst for each identified pathway. Criteria for pathway significance was strict for metabolomics and set as a Holm adjusted *p*-value < 0.05 and FDR < 0.01.

Paired Student’s *t*-test was used to compare average daily protein intake during pregnancy to postpartum, with *p* < 0.05 considered statistically significant.

## 3. Results

### 3.1. Characteristics of Subjects

Although 81 subjects were enrolled in this study, only the 47 women that completed both study days were included in this analysis. Their ethnicity was as follows: 74% White (*n* = 35), 9% Black (*n* = 4), 13% Asian (*n* = 6), 2% Hispanic (*n* = 1), and 2% Pacific Islander (*n* = 1). Subject demographics and laboratory tests can be found in Table 1. Average daily protein intake was not significantly different (*p* = 0.13) between pregnancy and postpartum (Table 1). Chylemia was not noted in any blood samples collected [17]. Metabolomic profiling identified 980 unique metabolites in the samples and all metabolite data can be found in Supplementary Files S1.

Semi-quantitative metabolomics analysis yielded relative peak area data for 980 unique metabolites. Of these, 706 were found to be significantly different between pregnancy and postpartum. Metabolomics analysis found three pathways to be significantly altered in pregnancy. After the identification of significant pathways and metabolites, 10 key AAs were selected for quantitative confirmational analysis. Concentrations and significance for these AAs were confirmed using quantitative LC/MS. Of the 10 selected metabolites, 7 were confirmed to be significantly different during pregnancy and all 10 matched the pregnancy/postpartum ratio found in the metabolomics analysis. These confirmational results allow for more direct conclusions to be drawn from the metabolomics data.

As expected, patient weight was significantly higher during pregnancy compared to postpartum (*p* < 0.05). Serum albumin, bilirubin, creatinine, and blood urea nitrogen were all significantly lower during pregnancy than postpartum (*p* < 0.05). Measured creatinine clearance was significantly higher during pregnancy than postpartum (*p* < 0.05).

### 3.2. Multivariate Analysis

**Figure 1:**
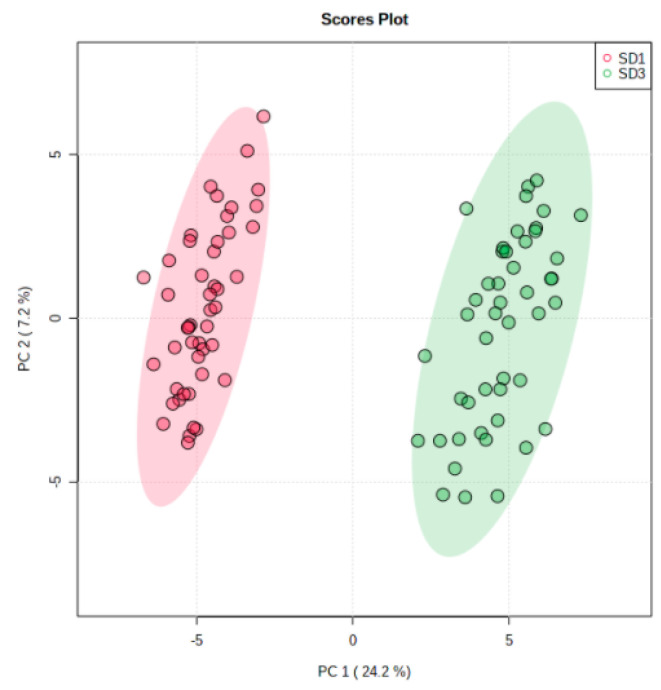
Principal component analysis (PCA) results for comparison of paired plasma samples from 47 pregnant (red) and postpartum (green) women. This aligns with the legend in the upper right where SD1 = study day 1 (pregnant) and SD3 = study day 3 (postpartum). The 980 identified metabolites were used to find the dimensions of greatest variation between the two groups with subjects serving as their own controls. Percent variance values explained by the principal components 1 and 2 are 24.2% and 7.2% respectively.

Unsupervised principal component analysis (PCA) was used to give a high-level view of the segregation and grouping of pregnant and postpartum plasma samples. This was performed for the 94 plasma samples from 47 individual participants, which demonstrated a loose segregation between the pregnant and postpartum study days. This is visualized by the coloring of groups in Figure 1.

### 3.3. Univariate Analysis

**Figure 2:**
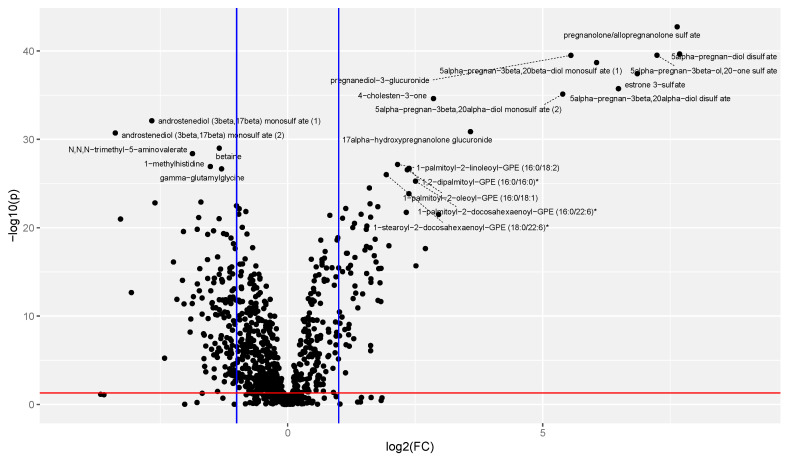
Volcano plot representing the magnitude and significance of changes observed with −log10(*p*-value) on the y-axis and log2(Fold Change (FC)) on the x-axis. The cutoff of the significance value of *p* < 0.05 is noted with the horizontal red line and the thresholds for fold change values of greater than 2 or less than 0.5 are noted with vertical blue lines. Each point represents a single metabolite. Metabolites with a −log10(*p*) > 25 are labeled with their chemical name.

The segregation between groups in Figure 1 demonstrates an overview of the large metabolic changes that take place during pregnancy. Figure 2 demonstrates the direction of significant changes identified by matched pairs *t*-test with a volcano plot.

Untargeted metabolomic pathway analysis was then performed. This resulted in the identification of three pathways (of the 64 present pathways) that are significantly impacted during pregnancy (*p* < 0.05 and FDR < 0.01). These included valine, leucine, and isoleucine metabolism (*p* = 2 × 10^−5^ and FDR = 1 × 10^−3^), caffeine/xanthine metabolism (*p* = 4 × 10^−5^ and FDR = 1 × 10^−3^), and arginine biosynthesis (*p* = 5 × 10^−5^ and FDR = 1 × 10^−3^). Aside from caffeine metabolism, all significant pathways are part of the amino acid metabolism, demonstrating a significant impact of pregnancy on the metabolism of many amino acids.

**Figure 3:**
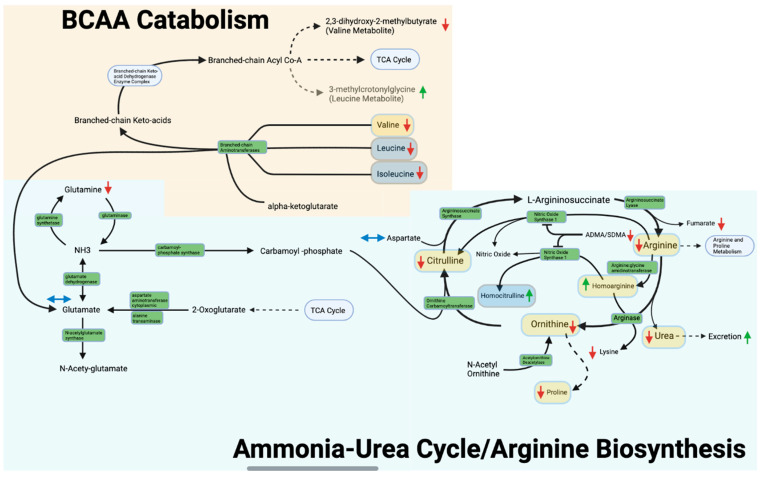
Arginine Biosynthesis, Ammonia-Urea Cycle and BCAA Catabolism. Enzymes are shown in green. Downward pointing arrows indicate lower metabolite peak areas during pregnancy (red), while upward arrows indicate higher peak area during pregnancy as compared to the non-pregnant postpartum control (green). Double pointed horizontal arrows indicate no change (blue). Metabolites that were subject to confirmatory quantitative analysis and confirmed to be significant are shown with rounded yellow translucent rectangles while those not significant in the confirmatory analysis are blue/gray. Asymmetric dimethylarginine and symmetric dimethylarginine are shown as ADMA/SDMA. Pathway structure was modeled using the Kyoto Encyclopedia of Genes and Genomes.

Using the Kyoto Encyclopedia of Genes and Genomes (KEGG) database (October 2021 version) substrates of the arginine biosynthesis and valine, leucine, and isoleucine metabolism pathways were identified. Of these we have included peak area data for 22 as shown in Table 2 and in Figure 3. Arginine biosynthesis and BCAA metabolism were presented in this paper in the context of pregnancy.

### 3.4. Arginine Biosynthesis and Ammonia-Urea Cycle

Table 2 includes mean normalized peak area data for the arginine biosynthesis metabolites and the results of the direct comparison of metabolite peak areas between pregnancy and postpartum (represented by pregnancy/postpartum ratios). Figure 3 depicts the effects of pregnancy on arginine biosynthesis, the ammonia-urea cycle, and BCAA catabolism. Arginine, ornithine, citrulline, proline, glutamine, urea, fumarate, N-acetylglutamate, and N-acetylarginine peak areas were all significantly lower during pregnancy than postpartum (Figure 3). In contrast, the peak areas were significantly higher during pregnancy for homoarginine and homocitrulline (Figure 3). The most altered metabolite of interest in both metabolomics and confirmational analysis was homoarginine with pregnancy/postpartum ratios of 2.49 and 2.41 respectively. Quantitative mass spectrometry analysis performed for the pathway amino acids arginine, proline, citrulline, ornithine, and homoarginine confirmed significant changes in plasma metabolite concentrations. Homocitrulline was not found to be significantly altered in the confirmational analysis after Bonferroni correction but met the *p* < 0.05 significance cutoff prior to correction (non-corrected *p* = 5 × 10^−2^). The confirmational ratio for homocitrulline still closely matched that found in metabolomics as shown in Table 3. A metabolic panel performed on each study day confirmed a significant change in serum urea concentrations.

The arginine biosynthesis pathway, including the urea cycle, was found to be significantly altered by pregnancy (*p* < 0.05 and q < 0.01), with an FDR < 0.001. This pathway includes the entire urea cycle as well as ammonia nitrogen disposition and glutamine deamination as seen in Figure 3.

### 3.5. Valine, Leucine, and Isoleucine Metabolism

Table 2 also includes peak area data for BCAAs (valine, threonine, leucine, and isoleucine) as well as BCAA metabolites (3-methylcrotonyl lycine and 2,3-dihydroxy-2-methylbutyrate) (Figure 3). Of these BCAAs and metabolites, valine, leucine, isoleucine, and 2,3-dihydroxy-2-methylbutyrate were shown in metabolomics analysis to be significantly lower (*p* < 0.05 and q < 0.01) in pregnancy while threonine and 3-methylcrotonylglycine were significantly higher. Targeted confirmational analysis confirmed significance and pregnant/postpartum ratios for valine and threonine (*p* < 0.05). Confirmation for leucine and isoleucine was not significant after Bonferroni correction but confirmational pregnant/postpartum ratios matched with those in the metabolomics analysis. Metabolomics analysis identified 2,3-dihydroxy-2-methylbutyrate as the most altered metabolite of the valine, leucine, and isoleucine metabolism with a pregnant/postpartum ratio of 0.46 as seen in Table 2.

The valine, leucine, and isoleucine metabolism pathway as a whole was also determined to be significantly altered by pregnancy (*p* < 0.05 and q <0.01), with an FDR < 0.001. This pathway includes the branched chain amino transferases as seen in Figure 3.

## 4. Discussion

### 4.1. Overview

This is the first study to assess the ammonia-urea cycle and BCAA metabolism using metabolomic pathway analysis with confirmatory quantitative analysis between pregnancy and postpartum, with each subject serving as their own control. This design was used to couple broad metabolomics with a higher-powered confirmatory analysis that directly validated analytes in significant pathways. These methods allow for more direct conclusions to be drawn from metabolomics data and strengthen the results of pathway analysis.

We found that pregnancy significantly impacts arginine biosynthesis; valine, leucine, and isoleucine plasma concentrations; as well as caffeine/xanthine metabolism. Of these three pathways, only caffeine/xanthine metabolism has previously been reported to be impacted during pregnancy [18].

AA concentrations have previously been measured in pregnancy and postpartum, but our methodology is unique as it includes validation with targeted assays. Our AA concentration findings were consistent with Kalhan et al. [6], Di Giulio et al. [3], and others, who observed lower plasma concentrations of leucine, valine, proline, citrulline, ornithine, and arginine and higher threonine concentrations as well as lower urea synthesis and BUN during pregnancy compared to postpartum [3,4,5,6,19]. Our study expanded on others with a significantly larger sample size (8 for Kalhan et. al. vs 47 for this study), using each subject as their own control, and the inclusion of more metabolites. Our results also align with the decrease in urea concentrations noted by Cheung and Lafayette, the decreased levels of NOS inhibitors ADMA/SDMA found by Holden et al., and the increase of NOS substrate and competitive inhibitor homoarginine demonstrated by Valtonen et al. [7,20,21].

### 4.2. Arginine Biosynthesis/Nitrogen Disposition

Our data show significantly lower plasma ammonia-urea cycle intermediates as well as the higher overall renal clearance typically seen during pregnancy [22,23].

Our data suggest these metabolic changes could be a result of the conservation of ammonia nitrogen through the limitation of AA deamination making less nitrogen available to be transferred by glutamine to the liver. Glutamine nitrogen transport represents one of the largest contributors of nitrogen to hepatic ammonia-urea metabolism [24]. Diet and glutamine synthesis from ammonia and glutamate are the only substantial sources of glutamine in the body, making amino nitrogen availability a key factor in glutamine synthesis [25]. We found that plasma glutamine concentrations were lower during pregnancy, which could lead to decreased availability of glutamine in the liver and less nitrogen availability to drive the ammonia-urea cycle. This reflects a conservation of amino nitrogen [26]. Such conservation aligns with the increased need for amino acids during pregnancy [27].

Lower concentrations of plasma glutamine and consequent metabolic limitation of nitrogen would also have downstream effects on the concentrations of ammonia-urea cycle intermediates. Carbamoyl phosphate synthase 1 (CPS1) is the rate-limiting step for the ammonia-urea cycle, which requires ammonia as a substrate [28]. With limited ammonia available from glutamine, the ammonia-urea cycle will lack a rate-limiting substrate decreasing urea turnover, and potentially leading to the observed decrease in levels of cycle intermediates. This is further supported by our confirmatory findings demonstrating a decrease in plasma levels of arginine and proline, whose de novo syntheses are dependent on ammonia-urea cycling [29].

Nitric oxide (NO) production is also connected to the ammonia-urea cycle with nitric oxide synthase (NOS) using arginine as a substrate. NO is a potent endogenous vasodilator that aids in blood pressure regulation and uterine vascular remodeling [30,31]. Both the main substrate of NOS (arginine) and the main product (citrulline) were found to be significantly decreased in pregnancy, possibly limiting their availability to NOS and impacting endogenous NO production. This has possible implications in changes in vascular resistance in pregnancy, including gestational hypertension and preeclampsia, as is further suggested by the preeclamptic symptoms recorded in animal models when NOS is inhibited [32,33]. Homoarginine and its precursor homocitrulline were found to be significantly higher in pregnancy. These AA analogs may serve as substrates for NOS in pregnancy when more typical sources are not available [34].

### 4.3. Valine, Leucine, and Isoleucine Metabolism

Our study also supports an alteration in BCAA metabolism. BCAAs cannot be synthesized de novo so any change in their concentrations during pregnancy must result from changes in either availability in diet, incorporation into protein, release from protein, or oxidative catabolism [35]. Our data illustrates a decrease in levels of the plasma BCAAs, particularly valine, during pregnancy with no significant change in average protein intake for the 3 days prior to each study day. This aligns with a decrease in urea production and conservation of amino nitrogen as discussed in 3.2, as lower concentrations of BCAAs allow for less BCAA catabolism and decreased nitrogen availability for urea synthesis. During pregnancy, the placenta and fetus have high AA uptake leading to increased disappearance of AAs from maternal plasma and greater AA concentrations in placental and fetal circulations [36]. This could account for the decrease observed in maternal plasma BCAAs. With BCAAs being essential to body protein mass and modulation of insulin growth factors in gestation, their presence is critical for fetal growth and healthy pregnancy [8,11,37]. Valine had the largest fold change in the BCAAs measured in metabolomics and LC/MS confirmatory analyses. Valine has implications in maternal glucose concentrations during pregnancy and changes in BCAA metabolism could contribute to gestational diabetes mellitus and other metabolic disorders during pregnancy [38,39,40,41]. Animal studies have shown BCAA supplementation to improve fetal growth and increase birth weight, but no comparable studies have been carried out in humans [37]. Two other notably perturbed pathway metabolites were 3-methylcrotonylglycine and 2,3-dihydroxy-2-methylbutyrate, both of which appear to be involved in BCAA metabolism [42,43]. Overall, there has been limited research on the specific role of BCAAs during pregnancy beyond their incorporation into proteins required for fetal growth.

### 4.4. Limitations

The use of metabolomic analysis limits the specific conclusions that can be drawn in this study, however, the addition of quantitative confirmatory analysis of key amino acids helped to validate our findings. Given the overall consistency between confirmatory and metabolomics analyses, there is strong evidence to support a perturbation of AAs and nitrogen disposition during pregnancy. A significant limitation of this study is that blood sample collection occurred 1 h after starting breakfast, which could impact branched chain amino acid concentrations. However, average protein intake for the 3 days prior to each study day was not significantly different during pregnancy compared to postpartum. The study was also limited by only evaluating the pregnant metabolome at a single point during pregnancy compared to postpartum. Future studies could explore the metabolome at multiple points during pregnancy to gain further insight on the effect of gestational age on AA metabolism during pregnancy. Lastly, we were unable to quantify every metabolite with potential influence over the ammonia-urea cycle. Future work could focus on enzyme expression and amino acid excretion.

## 5. Conclusions

Pregnancy significantly impacts the ammonia-urea cycle and BCAA metabolism, supported by pathway significance in preliminary untargeted metabolomics and confirmed significant changes in arginine, citrulline, homoarginine, ornithine, proline, threonine, and valine. Our results align with prior research on metabolic changes in pregnancy and provide new insight on the broader context of these alterations. The results support amino nitrogen conservation during pregnancy, likely to support fetal and placental growth. These metabolic changes could possibly contribute to the development of pathology during pregnancy for women at risk.

## Figures and Tables

**Table 1:** Characteristics of subjects during pregnancy and postpartum.

Characteristics	Pregnant (*n* = 47)	Postpartum (*n* = 47)	*p*-Value
Gestational Age (weeks) or Time Postpartum (weeks)	27.0 ± 1.3	15.0 ± 2.1	NA
Height (cm)	163.6 ± 16.8		NA
Weight (kg)	71.6 ± 10.6	67.4 ± 9.3	1 × 10^−5^
IBW (kg)	58.2 ±6.4		NA
BMI (kg/m^2^)	25.7 ± 3.2		NA
Albumin (g/dL)	3.6 ± 0.2	4.6 ± 0.2	9 × 10^−28^
Bilirubin (mg/dL)	0.5 ± 0.2	0.7 ± 0.4	7 × 10^−7^
Serum Creatinine (mg/dL)	0.5 ± 0.1	0.7 ± 0.1	9 × 10^−21^
BUN (mg/dL)	7.8 ± 1.9	14.0 ± 4.0	1 × 10^−14^
CrCl (mL/min)	194.3 ± 81.4	130.9 ± 25.6	3 × 10^−6^
Mean Protein Intake (g/day)	81.3 ± 26.7	73.9 ± 22.4	0.13

*p*-values from paired Student’s *t*-test, BMI calculated using pre-pregnancy body weight, BMI = body mass index, IBW = ideal body weight, BUN = blood urea nitrogen, CrCl = creatinine clearance.

**Table 2:** Pregnant and postpartum pathway and metabolite peak area data from metabolomics analysis.

Pathway			*p*-Value	FDR	Holm Adjusted *p*-Value
Valine, Leucine, and Isoleucine Metabolism Caffeine/Xanthine Metabolism Arginine Biosynthesis			2 × 10^−5^ 4 × 10^−5^ 5 × 10^−5^	1 × 10^−3^ 1 × 10^−3^ 1 × 10^−3^	2 × 10^−3^ 4 × 10^−3^ 4 × 10^−3^
**Arginine Biosynthesis Metabolite**	**Pregnant**	**Postpartum**	**Pregnancy/** **Postpartum**	***p*** **-Value**	**q-Value**
ADMA/SDMA	0.92 ± 0.16	1.15 ± 0.22	0.80	1 × 10^−8^	4 × 10^−9^
Arginine	0.80 ± 0.17	1.25 ± 0.23	0.63	2 × 10^−15^	2 × 10^−15^
Aspartate	1.23 ± 0.78	1.07 ± 0.40	1.15	6 × 10^−1^	7 × 10^−2^
Citrulline	0.82 ± 0.26	1.30 ± 0.35	0.63	9 × 10^−14^	6 × 10^−14^
Fumarate	0.90 ± 0.19	1.15 ± 0.26	0.79	8 × 10^−11^	4 × 10^−11^
Glutamate	1.08 ± 0.72	1.06 ± 0.43	1.02	3 × 10^−1^	3 × 10^−2^
Glutamine	0.93 ± 0.13	1.11 ± 0.16	0.84	1 × 10^−10^	5 × 10^−11^
Homoarginine	1.88 ± 1.04	0.75 ± 0.31	2.49	2 × 10^−17^	3 × 10^−17^
Homocitrulline	1.22 ± 0.55	0.95 ± 0.51	1.29	1 × 10^−3^	2 × 10^−4^
N-acetylglutamate	0.96 ± 0.20	1.13 ± 0.32	0.85	3 × 10^−4^	5 × 10^−5^
N-acetylarginine	0.78 ± 0.36	1.55 ± 0.66	0.50	3 × 10^−23^	1 × 10^−22^
Ornithine	0.68 ± 0.19	1.39 ± 0.32	0.49	3 × 10^−18^	3 ×10^−18^
Proline	0.86 ± 0.25	1.16 ± 0.34	0.74	3 × 10^−8^	1 × 10^−8^
Urea	0.80 ± 0.27	1.53 ± 0.46	0.52	1 × 10^−16^	2 × 10^−16^
3-amino-2-piperidone	0.79 ± 0.16	1.37 ± 0.37	0.58	5 × 10^−20^	1 × 10^−19^
**BCAA Metabolism** **Metabolite**	**Pregnant**	**Postpartum**	**Pregnancy/** **Postpartum**	***p*-Value**	**q-Value**
Isoleucine	1.00 ± 0.30	1.09 ± 0.23	0.92	3 × 10^−2^	5 × 10^−3^
Leucine	0.97 ± 0.32	1.11 ± 0.24	0.87	1 × 10^−3^	2 × 10^−4^
Threonine	1.25 ± 0.26	0.76 ± 0.21	1.64	3 × 10^−14^	3 × 10^−14^
Valine	0.92 ± 0.23	1.09 ± 0.21	0.84	8 × 10^−5^	2 × 10^−5^
3-methylcrotonylglycine	0.96 ± 0.49	0.81 ± 0.43	1.22	5 × 10^−2^	4 × 10^−3^
2,3-dihydroxy-2-methylbutyrate	0.66 ± 0.21	1.43 ± 0.41	0.46	3 × 10^−17^	5 × 10^−17^

Pregnant and postpartum values represent median scaled peak areas. Pregnancy/postpartum p values from paired *t*-test, pathway *p* value is pathway-weighted and false discovery rate (FDR) adjusted. Impact is determined from the centrality (number of node connections between pathway nodes) and the magnitude of change in peak area between the two conditions. Asymmetric dimethylarginine = ADMA and symmetric dimethylarginine = SDMA. *p*-values, q-values, and fold change for all identified metabolites can be found in supplementary XLSX file S2.

**Table 3:** Pregnant and postpartum plasma concentration data from targeted confirmatory analysis.

Metabolite	Pregnant (nM)	Postpartum (nM)	Pregnancy/ Postpartum	Bonferroni Corrected *p*-Value
Arginine	844 ± 208	1042 ± 239	0.82	9 × 10^−6^
Citrulline	0.15 ± 0.07	0.28 ± 0.12	0.54	2 × 10^−12^
Homoarginine	0.06 ± 0.04	0.02 ± 0.01	2.41	2 × 10^−7^
Homocitrulline	0.005 ± 0.003	0.004 ± 0.002	1.18	5 × 10^−1^
Isoleucine	280 ± 129	312 ± 82	0.90	1
Leucine	448 ± 206	524 ± 153	0.86	5 × 10^−1^
Ornithine	0.35 ± 0.17	0.78 ± 0.37	0.45	2 × 10^−11^
Proline	626 ± 21	871 ± 285	0.72	7 × 10^−5^
Threonine	800 ± 177	525 ± 126	1.49	2 × 10^−9^
Valine	821 ± 272	1030 ± 220	0.80	2 × 10^−3^

Pregnancy/postpartum *p*-values from paired Student’s *t*-test are Bonferroni adjusted to account for multiple comparisons.

## Data Availability

The metabolomics data generated for this study is available in supplementary materials as a XLSX file.

## Supplementary Materials

The following supporting information can be downloaded at: https://www.mdpi.com/article/10.3390/metabo13020242/s1, XLSX File S1: Metabolomics Data, XLSX File S2: Ranked *p* & q values, Document S3: Disqualifying Medications.

## References

[1] Soma-Pillay P., Nelson-Piercy C., Tolppanen H., Mebazaa A. (2016). Physiological changes in pregnancy. Cardiovasc. J. Afr..

[2] Wu G. (2010). Amino Acids: Biochemistry and Nutrition.

[3] Di Giulio A.M., Carelli S., Castoldi R., Gorio A., Taricco E., Cetin I. (2004). Plasma amino acid concentrations throughout normal pregnancy and early stages of intrauterine growth restricted pregnancy. J. Matern. Neonatal Med..

[4] Schoengold D.M., Defiore R.H., Parlett R.C. (1978). Free amino acids in plasma throughout pregnancy. Am. J. Obstet. Gynecol..

[5] Kalhan S.C. (2000). Protein metabolism in pregnancy. Am. J. Clin. Nutr..

[6] Kalhan S.C., Tserng K.-Y., Gilfillan C., Dierker L.J. (1982). Metabolism of urea and glucose in normal and diabetic pregnancy. Metabolism.

[7] Holden D.P., Fickling S.A., Whitley G.S., Nussey S.S. (1998). Plasma concentrations of asymmetric dimethylarginine, a natural inhibitor of nitric oxide synthase, in normal pregnancy and preeclampsia. Am. J. Obstet. Gynecol..

[8] Brosnan J.T., Brosnan M.E. (2006). Branched-Chain Amino Acids: Enzyme and Substrate Regulation. J. Nutr..

[9] Hutson S.M., Sweatt A.J., Lanoue K.F. (2005). Branched-chain amino acid metabolism: Implications for establishing safe intakes. J. Nutr..

[10] Hutson S.M., Fenstermacher D., Mahar C. (1988). Role of mitochondrial transamination in branched chain amino acid metabolism. J. Biol. Chem..

[11] Mogami H., Yura S., Itoh H., Kawamura M., Fujii T., Suzuki A., Aoe S., Ogawa Y., Sagawa N., Konishi I. (2009). Isocaloric high-protein diet as well as branched-chain amino acids supplemented diet partially alleviates adverse consequences of maternal undernutrition on fetal growth. Growth Horm. IGF Res..

[12] Shum S., Bs A.Y., Fay E., Moreni S., Mao J., Czuba L., Wang C., Ms N.I., Hebert M.F. (2021). Infant Dextromethorphan and Dextrorphan Exposure via Breast Milk From Mothers Who Are CYP2D6 Extensive Metabolizers. J. Clin. Pharmacol..

[13] Collet T.H., Sonoyama T., Henning E., Keogh J.M., Ingram B., Kelway S., Guo L., Farooqi I.S. (2017). A Metabolomic Signature of Acute Caloric Restriction. J. Clin. Endocrinol. Metab..

[14] Storey J.D., Bass A.J., Dabney A., Robinson D. (2022). Qvalue: Q-Value Estimation for False Discovery Rate Control. R Package Version 2.28.0. https://github.com/jdstorey/qvalue.

[15] Xia J., Wishart D.S. (2011). Metabolomic Data Processing, Analysis, and Interpretation Using MetaboAnalyst. Curr. Protoc. Bioinform..

[16] Han L.W., Shi Y., Paquette A., Wang L., Bammler T.K., Mao Q. (2021). Key hepatic metabolic pathways are altered in germ-free mice during pregnancy. PLoS ONE.

[17] Zhang Y., Zhou J., Zheng W., Lan Z., Huang Z., Yang Q., Liu C., Gao R., Zhang Y. (2016). Clinical, biochemical and molecular analysis of two infants with familial chylomicronemia syndrome. Lipids Health Dis..

[18] Handelman S., Romero R., Tarca A.L., Pacora P., Ingram B., Maymon E., Chaiworapongsa T., Hassan S.S., Erez O. (2019). The plasma metabolome of women in early pregnancy differs from that of non-pregnant women. PLoS ONE.

[19] Zhou T., Du S., Sun D., Li X., Heianza Y., Hu G., Sun L., Pei X., Shang X., Qi L. (2022). Prevalence and Trends in Gestational Diabetes Mellitus Among Women in the United States, 2006–2017: A Population-Based Study. Front. Endocrinol..

[20] Cheung K., Lafayette R.A. (2013). Renal physiology of pregnancy. Adv. Chronic. Kidney Dis..

[21] Valtonen P., Laitinen T., Lyyra-Laitinen T., Raitakari O.T., Juonala M., Viikari J.S., Heiskanen N., Vanninen E., Punnonen K., Heinonen S. (2008). Serum L-Homoarginine Concentration is Elevated During Normal Pregnancy and is Related to Flow-Mediated Vasodilatation. Circ. J..

[22] Kalhan S.C., Rossi K.Q., Gruca L.L., Super D.M., Savin S.M. (1998). Relation between transamination of branched-chain amino acids and urea synthesis: Evidence from human pregnancy. Am. J. Physiol. Metab..

[23] Denne S.C., Patel D., Kalhan S.C. (1991). Leucine kinetics and fuel utilization during a brief fast in human pregnancy. Metabolism.

[24] Haüssinger D. (1990). Nitrogen metabolism in liver: Structural and functional organization and physiological relevance. Biochem. J..

[25] Cruzat V., Macedo Rogero M., Keane K.N., Curi R., Newsholme P. (2018). Glutamine: Metabolism and Immune Function, Supplementation and Clinical Translation. Nutrients.

[26] Weiner I.D., Verlander J.W. (2013). Renal Ammonia Metabolism and Transport. Compr. Physiol..

[27] Elango R., Ball R.O. (2016). Protein and Amino Acid Requirements during Pregnancy. Adv. Nutr. Int. Rev. J..

[28] Pearson D.L., Dawling S., Walsh W.F., Haines J.L., Christman B.W., Bazyk A., Scott N., Summar M.L. (2001). Neonatal Pulmonary Hypertension: Urea-Cycle Intermediates, Nitric Oxide Production, and Carbamoyl-Phosphate Synthetase Function. N. Engl. J. Med..

[29] Wu G., Bazer F.W., Datta S., Johnson G.A., Li P., Satterfield M.C., Spencer T. (2008). Proline metabolism in the conceptus: Implications for fetal growth and development. Amino Acids.

[30] Lopez-Jaramillo P., Arenas W.D., Garcia R.G., Rincon M.Y., López M. (2008). Review: The role of the L-arginine-nitric oxide pathway in preeclampsia. Ther. Adv. Cardiovasc. Dis..

[31] Maul H., Longo M., Saade G., Garfield R. (2003). Nitric Oxide and its Role During Pregnancy: From Ovulation to Delivery. Curr. Pharm. Des..

[32] Summar M.L., Gainer J.V., Pretorius M., Malave H., Harris S., Hall L.D., Weisberg A., Vaughan D.E., Christman B.W., Brown N.J. (2004). Relationship between Carbamoyl-Phosphate Synthetase Genotype and Systemic Vascular Function. Hypertension.

[33] Deng A., Engels K., Baylis C. (1996). Impact of nitric oxide deficiency on blood pressure and glomerular hemodynamic adaptations to pregnancy in the rat. Kidney Int..

[34] Hrabák A., Bajor T., Temesi A. (1994). Comparison of Substrate and Inhibitor Specificity of Arginase and Nitricm Oxide (NO) Synthase for Arginine Analogs and Related Compounds in Murine and Rat Macrophages. Biochem. Biophys. Res. Commun..

[35] Cynober L.A. (2002). Plasma amino acid levels with a note on membrane transport: Characteristics, regulation, and metabolic significance. Nutrition.

[36] Cetin I., Ronzoni S., Marconi A.M., Perugino G., Corbetta C., Battaglia F.C., Pardi G. (1996). Maternal concentrations and fetal-maternal concentration differences of plasma amino acids in normal and intrauterine growth-restricted pregnancies. Am. J. Obstet. Gynecol..

[37] Teodoro G.F.R., Vianna D., Torres-Leal F.L., Pantaleão L.C., Matos-Neto E.M., Donato J., Tirapegui J. (2012). Leucine Is Essential for Attenuating Fetal Growth Restriction Caused by a Protein-Restricted Diet in Rats. J. Nutr..

[38] Bahado-Singh R.O., Syngelaki A., Mandal R., Graham S.F., Akolekar R., Han B., Bjondahl T.C., Dong E., Bauer S., Alpay-Savasan Z. (2017). Metabolomic determination of pathogenesis of late-onset preeclampsia. J. Matern. Neonatal Med..

[39] Gh B.F.N.M. (2018). Application of metabolomics to preeclampsia diagnosis. Syst. Biol. Reprod. Med..

[40] Bahado-Singh R.O., Syngelaki A., Akolekar R., Mandal R., Bjondahl T.C., Han B., Dong E., Bauer S., Alpay-Savasan Z., Graham S. (2015). Validation of metabolomic models for prediction of early-onset preeclampsia. Am. J. Obstet. Gynecol..

[41] Zhao L., Wang M., Li J., Bi Y., Li M., Yang J. (2019). Association of Circulating Branched-Chain Amino Acids with Gestational Diabetes Mellitus: A Meta-Analysis. Int. J. Endocrinol. Metab..

[42] Baumgartner M.R., Dantas M., Suormala T., Almashanu S., Giunta C., Friebel D., Gebhardt B., Fowler B., Hoffmann G.F., Baumgartner E. (2004). Isolated 3-Methylcrotonyl-CoA Carboxylase Deficiency: Evidence for an Allele-Specific Dominant Negative Effect and Responsiveness to Biotin Therapy. Am. J. Hum. Genet..

[43] Fitzsimons P.E., Alston C.L., Bonnen P.E., Hughes J., Crushell E., Geraghty M.T., Tetreault M., O’Reilly P., Twomey E., Sheikh Y. (2018). Clinical, biochemical, and genetic features of four patients with short-chain enoyl-CoA hydratase (ECHS1) deficiency. Am. J. Med Genet. Part A.

