# Supplementary material for: The Effects of Pregnancy on Amino Acid Levels and Nitrogen Disposition
Source: Metabolites. 2023 Feb 7;13(2):242. doi: 10.3390/metabo13020242 (PMC9961409; doi:10.3390/metabo13020242)
Supplement: Supplementary file 1 [file metabolites-13-00242-s001.zip › Disqualifying Medications.pdf]

Disqualifying Medications

abiraterone, almotriptan, amiodarone, amitriptyline, amoxapine, asenapine, bupropion, celecoxib, chloroquine, chlorpheniramine, chlorpromazine, cinacalcet, citalopram, clobazam, clorgyline, cobicistat, clomipramine, clozapine, codeine, darifenacin, darunavir, desipramine, desvenlafaxine, diphenhydramine, dolasetron, doxepin, dronedarone, duloxetine, eliglustat, escitalopram, fentanyl, flecainide, fluoxetine, fluvoxamine, granisetron, halofantrine, haloperidol, hydroxychloroquine, hydroxytryptophan, impramine, iproniazid, isocarboxazid, levomilnacipran, linezolid, lorcaserin, lumefantrine, meperidine, methadone, milnacipran, mirabegron, mirtazapine, moclobemide, nialamide, nortriptyline, palonosetron, panobinostat, pargyline, paroxetine, pazopanib, perphenazine, phenelzine, procarbazine, promethazine, propafenone, propoxyphene, protriptyline, quinidine, quinine, quinacrine, ranolazine, rasagiline, rifampin, risperidone, ritonavir, selegiline, sertraline, sibutramine, terbinafine, tramadol, trazodone, trimipramine, thioridazine, toloxatone, tranylcypromine, vemurafenib, venlafaxine, vilazodone, vortioxetine, ziprasidone
